# Disability inclusion and participation in Mount Frere, Eastern Cape: Barriers and facilitators

**DOI:** 10.4102/ajod.v14i0.1735

**Published:** 2025-10-31

**Authors:** Yanga Manxusa, Michelle Botha

**Affiliations:** 1 Department of Global Health, Division of Disability and Rehabilitation Studies, Faculty of Medicine and Health Sciences, Stellenbosch University, Cape Town, South Africa

**Keywords:** physical disabilities, rural communities, inclusion, participation, cultural beliefs, resilience

## Abstract

**Background:**

Rural settings may present particular challenges to the inclusion and participation of people with physical disabilities (PWPD). These relate to the physical environment, infrastructure and service delivery issues, socioeconomic constraints and specific traditional and cultural beliefs surrounding disability. Targeted interventions require an understanding of these contextual specifics.

**Objectives:**

This study investigated the lived experiences of people with disabilities in relation to social inclusion and participation in Mount Frere, a rural town in the Eastern Cape, South Africa.

**Method:**

A qualitative phenomenological approach was used to explore the barriers, facilitators and underlying cultural perceptions that shape the inclusion and participation experiences of PWPD in this community. Five participants with paraplegia were interviewed using semi-structured interviews, and thematic analysis was employed.

**Results:**

The findings reveal significant challenges, including societal stigma rooted in traditional beliefs, infrastructural inaccessibility and economic constraints, which collectively hinder social inclusion and participation. Despite these barriers, the participants’ resilience, adaptability and agency were evident. The results illustrate the importance of a biopsychosocial approach to understanding the barriers and facilitators to inclusion and participation for PWPD in rural settings.

**Conclusion:**

The study highlights the need for a holistic approach to disability interventions, emphasising development, community education to combat stigma and the promotion of economic empowerment for PWPD.

**Contribution:**

These findings contribute to the broader discourse on disability rights in rural South African contexts and call for targeted, context-specific policies to enhance inclusion and participation.

## Introduction

In South African rural settings, people with disabilities face particular barriers to social inclusion and participation (Dassah et al. 2018; Vergunst et al. 2017). Vergunst et al. (2017) highlight barriers to access to healthcare for people with disabilities in rural areas that include transportation challenges, accessibility challenges, discriminatory attitudes from health workers and communication challenges. Similarly, Vanderschuren and Nnene (2021) document barriers to free movement in urban spaces, such as inaccessible public transportation to wheelchair users, the lack of kerb cuts and ramps in most places and poor maintenance of sidewalks – barriers compounded in rural settings. While much of the literature has focused on barriers to healthcare access in rural settings, there is a need to investigate access challenges more broadly, including access to social and cultural life, economic opportunities and leisure and recreation (Cieza et al. 2018; WHO 2001; WHO et al. 2010). Importantly, investigating inclusion and participation experiences across various social spheres must recognise how both systemic barriers and societal perceptions create disabling circumstances.

Social inclusion and participation are integral to mental health and well-being (Chen et al. 2022; Gokmen et al. 2023). Yet, people with disabilities are often denied these opportunities – deemed unfit to participate by society based on a narrow and limiting understanding of the experience of impairments, capabilities and adaptations and people with disabilities’ interest to participate (Martin 2013). This is defined as ableism – discrimination against people with disabilities, which can be in the form of a thought, speech and action (Campbell 2009). Ableist discrimination rests on assumptions on what constitutes ‘normal function’ and links this to social value (Campbell 2009). In our societies, then, there is often an unspoken, underlying premise that the understandings of people without disabilities are more worthy or that those understandings are enough. This systemic silencing removes people with disabilities from the centre of conversations about disability access and inclusion, reinforcing narrow and often negative ideas about the nature, needs and capabilities of people with disabilities, including those with physical disabilities (Botha & Watermeyer 2024; Jones & Cheuk 2021).

This article presents the lived experiences of people with physical disabilities (PWPD hereafter) residing in Mount Frere, a small rural town in the Eastern Cape, South Africa. These experiences were gathered through a qualitative research study that explored how ableism operates in this rural setting, shaping the inclusion and participation of PWPD. The study acknowledges the impact of cultural beliefs, resource constraints and social structures as contextual factors in rural settings that intersect to strengthen or disrupt ableism. We hope to offer new insights into how ableism is lived and defied by PWPD. Giving a platform to people with disabilities is not only about representation; it is an issue of recognition of their agency, the subjugation of their lived realities and taking apart the structures that have worked to keep them silent (Smilges 2022).

Physical disability encompasses conditions that affect a person’s ability to perform activities, including walking, climbing stairs, lifting, reaching an object and carrying (McDougall, Wright & Rosenbaum 2010). However, as noted, social stigma attached to physical disabilities is arguably the central disabling factor that affects the social inclusion and effective participation and, by extension, the well-being of PWPD (Babik & Gardner 2021). This group is also particularly visible in the community, and this may attract particular forms of stigma (Garland-Thomson 2009).

Below, we begin by providing some description of the cultural and material contexts that PWPD are likely to have to navigate in rural settings in Africa, based on evidence from the literature. This is followed by a brief description of the conceptual lenses employed in this study, namely the Biopsychosocial Model of Disability. Here, we also define a crucial distinction between the concepts of ‘inclusion’ and ‘participation’.

### Context: Ableist barriers in rural settings

Customs, rituals and traditional beliefs form an integral part of indigenous rural communities in South Africa. These may also include and intersect with beliefs grounded in Christianity. Reviewing the literature on how disability is factored into these belief systems reveals divergent knowledge, a dichotomy of positive and negative cultural perspectives that people with disabilities must navigate. The latter relates to the view of disability as a punishment for wrongdoing, connected to the strong belief in ancestors (Donkor 2011; Ngubane-Mokiwa 2018), or as bewitchment, connected to anxieties about opposing spiritual forces (Ennion & Rhoda 2016). Ideas of disability as punishment for sin are also found in Christianity; for example, in Deuteronomy 28:27–29, we find that bodily ailments are listed as curses for sinfulness, further affirming perceptions of disability as the wrath of God.

These beliefs can negatively impact on health-seeking behaviours (Ennion & Rhoda 2016; Mhalu et al. 2019) and the efficacy of awareness programmes to promote disability inclusion. Moreover, communities may respond to anxieties about disability, curse and contagion by segregating community members with disabilities (Edomwonyi & Oniminya 2014). Scholars argue that actively incorporating traditional beliefs and health practices, and including traditional healers as healthcare stakeholders, is essential to promoting health-seeking behaviour in culturally coherent settings (Ohajunwa & Sefotho 2024).

On the other hand, scholars have noted the transformative potential of the concept of Ubuntu, an ethic distinct to Africa, for promoting disability inclusion and safeguarding the humanity and dignity of PWPD (Dwadwa-Henda, Mji & Ohajunwa 2025; Ned 2022). Ubuntu is premised on the notion that everyone is inherently valuable and interconnected with their community, challenging ableist perspectives (Ohajunwa & Mji 2021). Marovah and Mutanga (2023) further emphasise that Ubuntu is not merely a cultural value but a regenerative African framework for disability that emanates from care, solidarity and recognition of one another, countering Western individualism and reconceptualising inclusion grounded in interconnectedness. Dwadwa-Henda et al. (2025), for example, conducted a study in rural communities in South Africa, finding that people with disabilities are actively included in rituals. This inclusion is based on the belief in inherent and mutually reinforcing humanity, which transcends ability and vulnerability – as reflected in their study title, ‘The soul has no disability’ (Dwadwa-Henda et al. 2025). While Ubuntu offers a strong foundation for inclusion, scholars emphasise that it must be integrated with modern disability rights frameworks to ensure that individuals with disabilities can participate equally in all aspects of life (Ned 2022).

People with physical disabilities in rural contexts might be constrained in a world of contradictions, straddling malignant traditional views that connect disability to curse and contagion and supportive beliefs that safeguard humanity and dignity based on the ethic of Ubuntu. Evolving modernity and the colonial importation of Western values to Africa further complicate the scenario (Grech 2016). Materially, there are also resource constraints in rural areas that militate against inclusion. People with disabilities in rural areas are less likely to attend school, to be employed, to be attended by a healthcare professional and to own a cell phone and are more likely to be left behind in rural development initiatives (Jonckheere 2020).

### Conceptual framework: Biopsychosocial model and the inclusion and participation distinction

While this study adopted a phenomenological methodology to remain as close as possible to an individual’s experiences without directing the scalpel of the research agenda of the authors, ultimately the biopsychosocial model was used as a lens to read across the data. This was done to aid in the interpretation and discussion of the barriers and facilitators post-analysis.

It is crucial to understand that these barriers and facilitators involve not only biological factors related to health and impairment but also psychological health and social factors. The biopsychosocial model was conceptualised in 1977 by George L. Engel. Engel recognised the deficiency of the biomedical model of health and illness, which focused solely on biological factors affecting health, and the need for a more holistic approach to understanding the causes and remedies of illness and disability (Engel 1977). The resulting biopsychosocial approach emphasises how societal attitudes, physical environments and psycho-emotional well-being interface with biological conditions to promote or hinder good health and well-being outcomes (Wade & Halligan 2017). Understanding this interplay enables a more holistic exploration of the constitution of barriers and facilitators to social inclusion and participation and the consequences of these for individual PWPD.

Both the biopsychosocial model as a strategy and as a philosophy provide a helpful, holistic framework by bringing together the biomedical, psychological and social aspects of disability. However, it is worth considering the contextualisation of the model within Western epistemology. In rural African settings, where local knowledge systems, relational systems of belief and spirituality continue to shape the experiences of disability, such models may not necessarily reflect regional realities. The calls to decolonise African disability studies are, therefore, warranted, as they advance the acknowledgement and inclusion of African epistemologies, such as Ubuntu, which highlight community, interdependence and dignity as ways of thinking about disability. This would enrich the applicability of the biopsychosocial model by integrating African perspectives, which provide an interpretation of barriers and facilitators based on culture and context.

A further conceptual underpinning of this study relates to the distinction between ‘inclusion’ and ‘participation’. It continues to be difficult to define these terms independently, and inclusion is often cited in simplistic terms as the solution to exclusion (Davey & Gordon 2017). However, we cannot just replace exclusion with inclusion; we need to address the multi-layered barriers that people with disabilities face to ensure that they can participate (Botha et al. 2023). ‘Inclusion’ means the opportunity for all people to be welcomed into social spaces, free from unfair barriers and prejudice. It is the precursor for participation. ‘Participation’, however, involves the actual engagement of people in activities in a meaningful manner. Bowa (2024) asserts that disability inclusion in Africa must be rooted in Ubuntu and sustainable development based on the values of interdependence, dignity and addressing structural inequalities. It transcends mere physical presence and goes beyond issues of poverty and marginalisation, giving recognition to the engagement of those living with disabilities:

- Inclusion makes participation possible, and participation completes the goal of inclusion (Quick & Feldman 2011). It is therefore not enough that people with disabilities are able to occupy a space; they need to be able to take part in all activities of life on an equal basis with others, as enshrined in the United Nations Convention on the Rights of Persons with Disabilities (UNCRPD) (United Nations [UN] 2007). Bowa (2024) theorises the exercise of support for inclusion as a communal responsibility, which links the welfare of people with disabilities to the moral well-being of the entire community and questions an individualistic understanding of inclusion.

## Research methods and design

This study adopted a qualitative phenomenological approach. According to Lester (1999), phenomenological design is a research approach that examines how participants think about, interpret and experience specific phenomena in their daily lives. Phenomenological design is used in acquiring depth and critical understanding through focusing on participants’ understanding of a particular subject matter (Qutoshi 2018) – in this case, lived experiences of inclusion and participation.

### Sampling and recruitment

Mount Frere is a small remote setting. In the field of disability, there is no literature for this region. To utilise a qualitative phenomenology seeking rich data, the first author used purposive sampling, later followed by snowball sampling, to identify a sample of five PWPD. Though small, five participants can be a minimum for qualitative research (Dworkin 2012). Primarily, a purposive homogeneous sampling procedure was used. In this sampling method, participants must have similar traits or characteristics (Taherodoost 2016). A snowball sampling strategy was also used because the first sampling strategy was not entirely successful. A snowball sampling method is used when participants assist the authors in acquiring participants that fit the criterion (Taherodoost 2016). With their consent to act as intermediaries, initial contacts shared the study information with potential participants from their networks.

Participants were required to have the following characteristics for inclusion in the study:

- Participants were all 18 years and above, as this is the legal age for adulthood decisions.
- All participants were residing or have for most of their lives resided in Mount Frere.
- All participants were persons who are paraplegic, as this study focused specifically on the experiences of PWPD.

The exclusion criteria were as follows:

- People with multiple disabilities or other impairment types were excluded, as the study was focused on specific beliefs that surround PWPD and their resulting experiences of social inclusion and participation.
- People who have been living with a disability for less than 5 years were excluded, as they might not have in-depth experience in all aspects of life with regard to social inclusion and participation.

Recruitment was done from the first author’s network of contacts in the community, where he lives and works as a health professional. It is important to be clear that the potential participants were not the first author’s patients. He approached the participants in person and explained the study and asked if they were willing to consent as participants. Some accepted and some declined, without explanation; however, in tight-knit communities, privacy might be a reason for such non-acceptance.

In line with ethical research principles, care was taken to ensure voluntary participation without any form of coercion (Nelson et al. 2011). Potential participants were explicitly informed that their decision to participate or decline would have no impact on their relationship with the first author or their access to any services, and they were free to withdraw from the study at any time without consequences.

### Participants

The study included five participants, and each was assigned a pseudonym. The following section provides brief demographic and contextual profiles of each participant to help readers understand their diverse backgrounds and experiences while maintaining confidentiality. These descriptions offer an important context for interpreting their perspectives on disability and social inclusion and participation.

Mr. Simon Ceba (mid-50s) was employed in a senior management position prior to the accident, which rendered him paraplegic. He was a married man with several children. He was known to be tall, muscular and strong – a status that, coupled with his professional and social roles, commanded great respect. After the accident, his previous employment enabled him to retire comfortably, with medical aid schemes that continue to support his access to private healthcare, even if it is over 100 km from his residence. However, the accident damaged his personal life. His wife abandoned him and moved away, taking his grown-up children.

Mr. Ali Harrison (58 years old) became paraplegic at the age of 5. The cause has never been properly determined. His growing-up years in a rural area involved trying to navigate life in a wheelchair. Although he enjoyed sporting and social activities, the necessity for being assisted discouraged him from taking part, as he never wanted to be a burden on other people. He has never had a romantic partner, and he has no children. He lives with his family and runs a small shop from their one-roomed residence.

Mr. Kenny Jojo (66 years old) was paralysed in an accident 8 years ago. He lives with his wife and grandchildren. Though he never had formal employment, he used to provide for his family through manual labour. Now, being unable to work, he relies on a state disability grant.

Ms. Angelina Sithole (24 years old) experienced a car accident when she was 18, which left her paralysed. Prior to that, she had been a student and a content creator on social media with quite a bubbly personality. She transferred to a distance learning course to continue her studies, and she depends on a state disability grant. She shies away from friends inviting her out and says that her interest in social media has faded.

Ms. Mqoqi (52 years old) was shot in the lumbar region, which left her paraplegic. She was married but got divorced after 15 years, on the day she received the news of being paraplegic. Further to this, her ex-husband received 50% of her pension funds. She has medical aid for her medical needs, as she is working as an administrator.

### Data collection

Semi-structured interviews were conducted, which enabled participants to expand on specific topics and explore areas that the first author may not have anticipated (Gibson & Brown 2009). The semi-structured interview guide that was developed was used to facilitate dialogue but left space for the participant to steer the flow and to be led by the passage of their own narratives. The guide, although based on the study’s aims and reviewed literature, served as a conversational aid only. As a result, participants’ lived experiences of ableism culture, material circumstances and social processes were made visible within a familiar rural context, in ways that we hope were respectful and organic.

Interviews were conducted in Xhosa, the first language of the participants and the first author. Interviews were recorded with participant consent, then transcribed in Xhosa and finally translated into English. The first author used a research assistant to check and affirm his translations as a means to ensure accuracy.

### Ethical considerations

The study received ethical clearance from the Stellenbosch University Health Research Ethics Committee on 26 March 2024 (HREC Reference No: S23/10/259). In accordance with cultural respect and norms, permission to conduct the study was requested at KwaBhaca Royal Kraal.

All participants were briefed on the study and were asked to sign an informed consent form stating that their participation was voluntary, that they understood their right to withdraw and that the first author had permission to record and transcribe their interview. Project information was provided in Xhosa, the predominant local language in the area. In addition, the participants’ names were anonymised to protect their privacy. The recorded data were securely stored. Participants were protected from any form of discomfort, be it physical or mental. Interviews took place at their preferred time and place. In case of emotional distress, the first author arranged for local social workers to provide counselling support. This safeguard was, however, not required.

#### A note on positionality

The first author was born and grew up in this region. He is also a private practice managing director located in this small town. He is widely known in this neighbourhood as a healthcare provider and as a child, brother, friend and tutor to some potential participants. He is not related to them by blood, but he is part of this community with strong African values. The recruitment was done in the community by one of their own, who was brought up as they were. Although the first author’s long-standing community presence helped establish rapport and trust in the recruitment and interview process, this dual role of the health professional and researcher may have also been a limitation. Some potential participants might have felt that the study was too directly connected to their therapeutic or personal history, leading them to prefer not to participate (Trondsen & Sandaunet 2009). Such tensions were considered in the study design, and the importance of stressing voluntary participation and confidentiality was taken into account.

### Data analysis

The study adopted inductive thematic analysis (Braun & Clarke 2006). This technique enabled the first author to allow the participants to narrate the stories of their inclusion and participation, bracketing his own ideas. Six phases of analysis were undertaken, which are:

- familiarisation with the data through transcribing, translating and reading the transcripts
- identification of items of potential interest through re-reading the data
- starting to produce primary codes
- grouping data fragments with similar meanings
- generating initial themes from these primary codes and reviewing the initial themes to ensure their distinctness from each other
- naming and defining themes.

Several means were undertaken to enhance the trustworthiness of the analysis. Firstly, the transcripts (which were translated) underwent back translations by a language translation expert to check for accuracy and cultural relevance. Secondly, a second coder was trained on the initial coding and theme generation, who also served as the study supervisor. This supported checking interpretation slippage and extending the boundaries of the theme to achieve reliability. Thirdly, transcripts were returned to the participants for validation to ensure that their views were indeed captured. This process of member checking provided credibility and confirmability to the data interpretation.

## Results

Table 1 provides an overview of the themes and sub-themes that emerged in the analysis.

**TABLE 1:** Themes and sub-themes.

Themes	Sub-themes
1. Social dynamics	1.1 Family dynamics 1.2 Cultural and social beliefs
2. Physical barriers	2.1 Accessibility and mobility 2.2 Economic implications or independence
3. Biopsychosocial implications	3.1 Preference for solitude 3.2 Personal loss, adaptation and self-reliance

### Theme 1: Social dynamics

Participant narratives demonstrated that community attitudes, influenced by traditional beliefs and sociocultural norms, act as barriers or facilitators to engagement in social activities. This is connected to the sense of belonging that individuals are able to foster, with emotional and psychological consequences.

#### Sub-theme 1.1: Family dynamics

The closest structure we have is the family. Participant experiences illustrate the emotional and psychological effects that result when family dynamics are disrupted by the onset of impairment:

‘Honestly, not that my marriage was perfect, but after the accident, things went worse’. (Mr. Ceba)

Similarly, one participant illustrated the dramatic change in family responses after the disability:

‘After the incident, no one cares for me’. (Mr Jojo)

Likewise, another participant shared:

‘My husband divorced when he discovered I will not walk again’. (Ms Mqoqi)

These participants expressed a sense of abandonment and the loss of their familial roles, which has been identified as central to disability-related trauma (Watermeyer & Swartz 2016).

Another participant’s experience also reflects abandonment, but this time from her social circle, as she shared:

‘Yes, my high school friends. I tried reaching out to them, but they just ignored me. Very sad. Ey, we were so close to each other.’ (Ms Sithole)

In contrast to the older participants, Ms Sithole described receiving more familial support, which is very important to counteract the rejection she describes. She described feeling secure when at home. It may be the case that older people with more familial responsibility experience greater disruption of their familial belonging during the onset of impairment.

#### Sub-theme 1.2: Cultural and social beliefs

Participants described the attitudes they encounter in the community, which appear to be based on a negative view of disability as holding no social value and as connected to moral transgressions (Donkor 2011; Ngubane-Mokiwa 2018). One participant, for instance, said:

‘There are certain things that the birth of a disabled child is culturally associated with. If, for example, two related individuals slept together and conceived, there is a high likelihood that the child will bear a certain form of disability.’ (Mr Ceba)

Such beliefs can further the stigmatisation of people with disabilities, which, after all, is quite often seen through the lens of superstition instead of understanding and empathy (Ennion & Rhoda 2016).

Participants described being socially ignored and isolated:

‘In my view and experience, people without disability ignore people with disability’. (Mr Harrison)

Mr. Harrison has been disabled from a young age and suggests here a lifetime of feeling overlooked in his community. Similarly, another participant said:

‘It is the attitude, and it persists. People would want to be dissociated with you’. (Mr jojo)

Interestingly, one participant gave a more optimistic account, saying:

‘I have never been discriminated in any form’. (Ms Sithole)

This contradicts the negative experiences of the other participants. A factor here might be age and gender. The older and male participants arguably have more social status and power to lose, which might cause them to experience stigma more keenly. In addition, Ms. Sithole’s strong support and care within her family (as seen earlier) may contribute to her more positive experience.

### Theme 2: Physical barriers

Participants identified a key barrier to inclusion and participation as being the failure of the government to improve infrastructure, including roads, water and shelter. Their accounts highlight not only physical barriers but also how these impact their well-being and sense of community belonging and cause economic constraints. Additionally, respondents indicated that community perceptions also limit their involvement:

‘People will also want to disassociate with you and I am not seen at all with the state I am in’. (Mr Harrison)

They point to a culture that too often ignores or stigmatises people living with disabilities.

#### Sub-theme 2.1: Accessibility and mobility

Participants recognised that the environment is to blame for their access challenges. One participant explained:

‘I am on a wheelchair … The landscape does not allow this wheelchair to move around’.

Similarly, another participant shared how navigating the inaccessible landscape could leave him vulnerable and prohibited him from participating in enjoyable community events:

‘I would take the longest route because there is uneven terrain here, and the roads are not wheelchair friendly. So, I woke up early and took a longer route, which is sometimes unsafe because of the thieves who would see a vulnerable being. I love events. Like I love music events, I enjoy many things but cannot attend because of the prohibiting factors.’ (Mr Harrison)

Furthermore, participants recognised that their rural setting also had implications for access to key services, as one participant shared:

‘The clinics are very far. They are not centralized’. (Mr Ceba)

These experiences align with similar studies into rural access challenges of PWPD (Dassah et al. 2018; Vergunst et al. 2017).

In addition, participants all shared the challenges they face in using public transport, specifically minibus taxis. These are similar to findings from other studies, which cite lack of space, and difficulty transferring from wheelchair to vehicle (Vergunst et al. 2015). One of the participants described the following:

‘I use taxis. One of the challenges is getting off my wheelchair and into the taxi and where to fold and place my wheelchair’. (Ms Sithole)

There are also social dynamics at play as participants navigate the inaccessible environment. For instance, one participant described being made to feel like a burden and an inconvenience by fellow commuters:

‘After the divorce, it was difficult for me to move around, especially using public transport; I would hear people complaining that they were late when the taxi stopped for me.’ (Ms Mqoqi)

It is therefore necessary to recognise the reinforcing relationship between physical access and social attitudes in shaping the inclusion and participation possibilities of PWPD (Martin 2013).

#### Sub-theme 2.2: Economic implications or independence

Physical barriers cause economic constraints, which further limit opportunities for participation. One participant described the added cost he faces due to travelling with a wheelchair:

‘Remember, it has financial implications; I will have to pay for three spaces – myself, my assistant, and the wheelchair’. (Mr. Jojo)

Similar experiences of accessing public transport have been captured by other studies (Vergunst et al. 2015).

Inaccessible infrastructure also impacts on independence and autonomy in managing one’s finances. As one participant explained about his disability grant payments:

‘No, I do not [*withdraw the money myself*]; I send someone I trust to withdraw money for me’. (Mr Harrison)

The physical inaccessibility of grant collection points creates a complex web of relational dependencies. When Mr. Harisson says he must ‘send someone I trust’, it reveals how infrastructure barriers force people with disabilities into potentially vulnerable situations.

Nevertheless, participants described pursuing economic and educational opportunities. One of the participants shared:

‘I have a small spaza shop selling chips and a few things. My challenge is the space I use, which is the same room I am sleeping in’. (Mrs Harrison)

While the challenge of managing personal and public space might be shared by others in this community, we can assume that running a small business from their sleeping room presents unique challenges for PWPD. Similarly, a participant’s sentiment shows the desperation for self-reliance despite physical and economic barriers:

‘So, even now, I force myself to do things for myself. I do not want to stay without doing anything. Hence, I am studying against all odds.’ (Ms Sithole)

While this kind of resilience is admirable, we must consider the physical and emotional toll that it might take. Scholars argue that society’s expectation for disabled people to demonstrate strength and adaptability can deny them the right to process grief and acknowledge limitations (Watermeyer & Botha 2025). This emerges further in theme 3.

### Theme 3: Biopsychosocial implications

The biopsychosocial model highlights the psychological toll of societal attitudes and physical exclusion on PWPD (Wade & Halligan 2017). It enables us to think beyond the material implications of ableism.

#### Sub-theme 3.1: Preference for solitude

Several participants shared that they preferred to withdraw from social life:

‘I lost interest in life as a whole. I do not want to be seen in public anymore’. (Mr Ceba)

Recall Mr. Ceba’s status as a venerated community member before his accident in contrast to his desire not to be seen, perhaps as a diminished version of himself. Similarly, another participant shared:

‘I wish I could disappear, and no one would see me’. (Ms. Mqoqi)

These extracts demonstrate the damage that can be done to an individual’s sense of belonging and self, not due to impairment affects alone but due to encountering social stigma (Watermeyer & Swartz 2016).

Another participant demonstrated a similar sentiment, coupled with a striving towards independence:

‘I love living my life lonely and not burdening people. Even now, I force myself to do things for myself’. (Ms Sithole)

It is worth considering the shift from a bubbly student with an active presence on social media to a person who ‘loves’ loneliness. Scholars have suggested that, to escape stigma, it is often necessary for people with disabilities to project maximum resilience and independence regardless of the physical or emotional cost this may demand (Watermeyer & Botha 2025). We should perhaps question to what extent isolation is a choice versus a necessity for these participants.

#### Sub-theme 3.2: Personal loss, adaptation and self-reliance

The participants spoke about the psycho-emotional adaptation that they needed to undergo after becoming disabled. One participant described a process of denying what was in reality his new norm:

‘I did not immediately accept that I am indeed permanently paralysed. I had hope that my functionality would be restored sometime in the near or distant future.’ (Mr Ceba)

This is a common response to experiencing impairment (Watermeyer & McKinney 2022). Scholars suggest that newly impaired persons need to adapt not only to a new physical reality but also to a new sense of the world around them, their community and their place within that community (Watermeyer & Botha 2025). This is further demonstrated by Mr. Jojo, who reflected on finding himself in a ‘compromising and useless state’, in contrast to his former role as a provider for his family.

One participant echoes a sense of loss and a process of grieving and remaking her self-concept:

‘Sometimes I feel useless and helpless being unable to do things that I used to do and enjoy … knowing I cannot do what was once pleasurable hurts’. (Ms Sithole)

This is counterbalanced with a demonstration of resilience and proactivity, as we have seen throughout Ms Sithole’s narrative, as she said: ‘Hence, I am studying against all odds’. This implies that the experience of loss is profound, but individuals can find their own adjustments while navigating what she calls their ‘path in life’, thereby demonstrating that personal loss does not always have to spell complete disempowerment.

## Discussion

The participants’ accounts emphasise the intersection between physical and social barriers, which shape everyday personal experiences (Martin 2013). They suggest that disability in rural contexts is complex and multifaceted due to context-specific societal attitudes, inadequate infrastructure and the lived experience of loss, which accompanies the destabilisation of social and familial roles (Watermeyer & Swartz 2016). These elements form intersecting systems that contribute to the ongoing marginalisation of PWPD.

Families often serve as the primary support system when individuals face distress, but disability can strain familial relationships, especially where pre-existing conflicts exist (Watermeyer & Swartz 2016). The entrance of disability can emotionally and psychologically strain family life, leading to feelings of betrayal and abandonment. However, Ms. Sithole’s narrative offers a more hopeful view of family dynamics. Her emphasis on homecoming reflects a deep sense of belonging within her family, contrasting her feelings of exclusion from the broader society. These varied responses demonstrate that the impact of disability on family interactions is complex and contingent upon individual circumstances and roles.

Negative experiences in the family are often reproduced in broader community life where disability can be associated with moral failure (Donkor 2011; Ngubane-Mokiwa 2018). Such beliefs reinforce harmful stereotypes, perpetuating stigma and marginalisation and leading to isolation for PWPD (Babik & Gardner 2021). However, experiences of disability-related stigma are not uniform. Ms. Sithole, for instance, did not perceive any discrimination against her. Experiences are therefore not monolithic and should not be assumed to be the same.

Physical barriers exist in a reinforcing relationship with these familial and social dynamics. In rural areas, there are particular access challenges due to the terrain, inadequate infrastructure and remoteness, meaning that facilities are often a distance away (Dassah et al. 2018; Vergunst et al. 2017). People with physical disabilities are often in vulnerable situations as a result, facing risks to their safety and health and encountering negative and belittling attitudes of the community that may view them as burdensome. Physical barriers go beyond this, though, as they also have significant socioeconomic consequences.

Lack of accessible infrastructure exacerbates social isolation, limits participation in community activities and limits opportunities for income generation, both formal and informal (Mitra & Palmer 2023). The economic challenges associated with physical disabilities are pronounced. Many PWPD face additional costs related to their disability, such as specialised transportation or home modifications. Some extra expenses can be viewed as inherently ableist, such as the requirement to pay to take a wheelchair onto public transport (Vergunst et al. 2015). Despite these challenges, there are also examples of agency and resilience among PWPD. Some, like Mr. Harrison, have developed strategies to achieve a degree of financial autonomy, illustrating the importance of self-dependence and flexibility in overcoming economic barriers.

The impact of disability extends beyond the interaction of physical impairment with the environment to include psychological and social dimensions (Wade & Halligan 2017). Each participant had undergone a social transition as a result of their impairment, causing them to make certain choices about how to interact with the community (Watermeyer & Botha 2025). Several described seeking solitude to cope with this transition, to avoid stigma, pity and discomfort in social situations. For example, Mr. Ceba and Ms. Mqoqi described their desire to retreat from the public gaze. This response echoes Engel’s (1977) view of the psychological cost of living with a disability in societies that are not fully accepting.

Isolation is often a rational response to stigma on the part of PWPD. However, coping strategies are influenced by factors such as the availability of social support networks and accessible facilities and services. Gondwana and Stewart (2013) emphasise that societal attitudes and environmental exclusion have a significant psychological impact, which aligns with the participants’ preference for solitude as a coping mechanism.

Part of providing social support may be to enable individuals to process personal loss (Watermeyer & Botha 2025; Watermeyer & Swartz 2016). Participants shared the sense that the onset of their impairments represented a significant loss to them. Loss is seldom discussed in ways that are transformative for PWPD (Watermeyer 2013). It is suggested that to move through loss into life with disability, individuals need to be enabled to speak about and share their emotions related to impairment and disability (Watermeyer & Botha 2025). Equally, people with disabilities’ right to inclusion and participation on an equal basis with others must be safeguarded. Botha et al. (2023) assert that seeking to address disability inclusion without properly considering the root causes of exclusion, such as stigma and resource inequities based on ableist constructions of disability, is superficial.

### Recommendations

These are implications from the participant experiences that portray the challenges that PWPD in rural areas go through daily, physically, socially, economically and emotionally:

- Infrastructure development: The need for better accessibility in public places emerged as a paramount concern among the participants. This finding reflects other studies (Dassah et al. 2018; Ennion & Rhoda 2016; Vergunst et al. 2017). Targeted infrastructure improvements are urgently needed. Local authorities should ensure infrastructure improvements, including wheelchair-friendly pathways and accessible public buildings. Furthermore, accessible public transport should be developed. There is also a need to root infrastructure development in community awareness on the rights and community contribution of PWPD.
- Community education: The study brought to light lingering negative cultural beliefs and stigma towards people with disabilities, causing experiences of isolation, abandonment, rejection and exclusion. This justifies the recommendations by Ned (2019) and Ohajunwa and Sefotho (2024) on the need to infuse indigenous knowledge and culture into health-related curricula. Given the cultural significance of traditional healers in rural areas, train them to advocate for inclusion and challenge harmful beliefs about disability, bridging traditional and modern approaches to inclusion. Broader community awareness programmes, especially for the young, are also recommended to foster inclusive behaviour from an early age, breaking the cycle of stigma in future generations. These efforts should ideally involve collaboration among community leaders, health professionals, traditional healers and, crucially, PWPD. The Ubuntu ethic should be utilised as a lens through which communities can view disability and the value of people with disabilities in community life (Dwadwa-Henda et al. 2025).
- Family support: The family is a central pillar of support for PWPD but can experience strain. Support for the whole family is required, and it is important for realising the rights of the PWPD (Mji et al. 2009; Naidoo & Ennion 2018). Family support services could include forming support groups, offering counselling services and providing practical skills training on caregiving, care for the carer and on disability and social justice.
- Economic empowerment: Programmes promoting economic independence are needed. Naidoo and Ennion (2018) note that in rural areas in South Africa, PWPD are often held in a cycle of ‘disability, immobility and poverty’. Interventions such as vocational training, microfinance initiatives, support for entrepreneurship and economic support for families are needed to break this cycle. It is important that these initiatives are responsive to local economic demand and contextual factors. They should be designed in collaboration with local business and PWPD to meet both market and individual needs. It is also important that existing rural economic development programmes include access for PWPD (Grech 2016). Intersectional factors related to gender, age, and community status and role should be considered (Ohajunwa & Mji 2021). It is also recommended that future studies be directed at assessing the effectiveness of such interventions and investigating their applicability in various rural contexts of South Africa.

## Conclusion

This study portrays the serious challenges of infrastructural barriers, persistent cultural stigma and economic hardships in the way of full social inclusion and participation. Yet, it simultaneously uncovers the resilience and adaptability of PWPD to surmount these hurdles with determination and, in many instances, find ways of asserting their independence and contributing to their communities. This study, therefore, shows the need for a holistic approach, which is essentially biopsychosocial, in understanding and addressing disability issues in rural settings in South Africa. A central conclusion is that policies and interventions that affect the lives of people with disabilities in these settings must be anchored around their voices and experiences, as well as responsive to geographical, socioeconomic and cultural contexts (Ohajunwa & Mji 2021). These findings suggest that in the development of policies and interventions around disability, local cultural beliefs and practices are pivotal. The study indeed brought to light how such traditional beliefs of disability may firmly bear upon the level of social inclusion and participation, raising the need for culturally sensitive initiatives meant to raise awareness about and foster inclusivity towards PWPD.

The results also affirm the applicability of the biopsychosocial model to research on disability in rural settings, as discussed by Wade and Halligan (2017). Considering the interaction of physical barriers, social attitudes and personal psychological responses, this study has demonstrated how the experiences of people with disabilities can be complex and multifaceted. It further builds on the work of Mitra and Palmer (2023), who explored disability and labour market outcomes in developing countries. This study depicts how factors such as gender, age, economic status and geographical location intersect with disability to create uniquely different challenges and experiences for PWPD in Mount Frere. It reinforces the necessity of prioritising the individual voices and stories of people with disabilities in research and particularly in contextually diverse rural settings (Sadiki, Watermeyer & Abrahams 2021).

The limitations of the study include: While the study has given rich data on individual experiences in-depth, it cannot represent the complete experiences of PWPD in Mount Frere or other rural settings because of the small number of participants. This limitation is particularly relevant given the diverse nature of disabilities and the different socioeconomic backgrounds represented in the region. The fact that this study only focused on one rural town limits its universality. Sadiki et al. (2021) clarify that experiences can vary significantly across rural settings. Mount Frere’s specific cultural, economic and infrastructural features may not be characteristic of other rural areas in and outside South Africa. Moreover, the absence of comparative data from urban settings or other rural areas raises limitations in distinguishing those aspects of experiences that are unique to Mount Frere and those that are more widely generalisable. These further raise the importance of multi-pronged investigations across different settings, rural and urban, as an attempt towards comprehensive insights into the experiences of PWPD in South Africa.
